# Total arch replacement for the enhanced-fibrinolytic-type disseminated intravascular coagulation patient with endoleak after thoracic endovascular aortic repair for aortic dissection

**DOI:** 10.1186/s44215-023-00046-1

**Published:** 2023-06-12

**Authors:** Ryo Kanamoto, Takeshi Oda, Keiichi Akaiwa, Katsuhiko Nakamura, Eiki Tayama

**Affiliations:** 1 Division of Cardiovascular Surgery, Omura Municipal Hospital, 133-22 Kogashima-Cho, Omura-Shi, Nagasaki, 856-8561 Japan; 2 Department of Cardiovascular Surgery, Department of Surgery, Kurume University School of Medicine, Kurume, Fukuoka Japan

**Keywords:** Disseminated intravascular coagulation, Endoleak, Total arch replacement

## Abstract

**Background:**

Endoleaks after stent graft treatment can cause disseminated intravascular coagulation (DIC), leading to a bleeding tendency.

**Case presentation:**

A 69-year-old man received thoracic endovascular aortic repair (TEVAR) for acute type B aortic dissection. After that, he developed bleeding tendency, and the diameter of his distal aortic arch increased. We diagnosed him with enhanced fibrinolytic-type DIC associated with a type Ia endoleak. We decided to perform a total arch replacement for the endoleak closure. To reduce the risk of massive bleeding, transfusion of fresh frozen plasma and platelets, oral tranexamic acid, and intravenous recombinant human soluble thrombomodulin were administered in the perioperative period. According to the multidisciplinary approach, the DIC improved, and the patient recovered.

**Conclusion:**

We successfully treated an endoleak-related DIC patient with bleeding tendency and combined correction for coagulopathy with supportive treatments.

## Introduction

Disseminated intravascular coagulation (DIC) is a life-threatening clinical condition, which provokes organ dysfunction and bleeding tendency [1]. Patients with DIC generally have some underlying diseases. Chronic DIC is sometimes observed in patients with aortic aneurysms or aortic dissection. Recently, persistent endoleaks following endovascular aneurysm repair (EVAR) are known to cause DIC [2–4].

We report a case of DIC associated with type Ia endoleak after thoracic endovascular aortic repair (TEVAR) for acute type B aortic dissection (TBAD). We treated the patient successfully with a combined total arch replacement (TAR) and medical control for DIC.

## Case

**Fig. 1:**
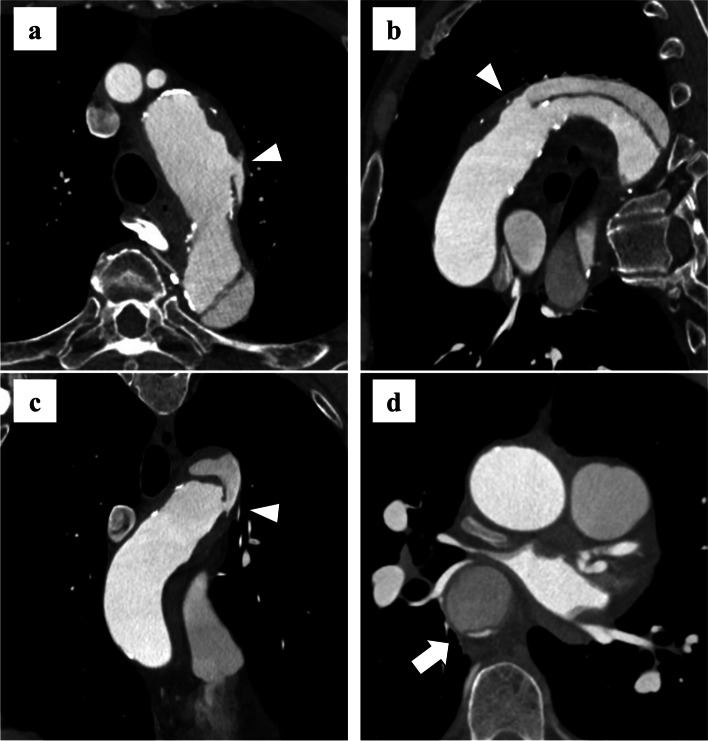
Contrast-enhanced computed tomography before thoracic endovascular aortic repair. Axial and multi-planar reconstruction (MPR) imaging reveals an acute type B aortic dissection. **a**–**c** The arrowheads indicate the primary entry located at the distal aortic arch. **d** The white arrow indicates the true lumen of descending and thoracoabdominal aorta severely compressed by the false lumen

**Fig. 2:**
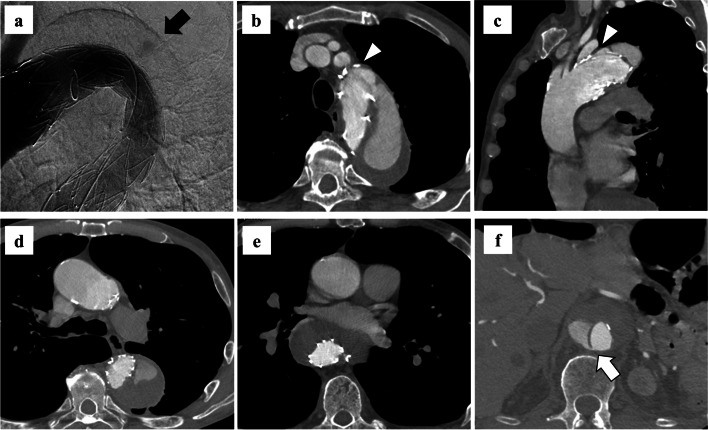
**a** Intraoperative digital subtraction angiography after deployment of the stent graft. The black arrow indicates endoleak. **b**–**f** Contrast-enhanced computed tomography after thoracic endovascular aortic repair. The false lumen remained open, and the diameter of the distal aortic arch was enlarged. **b**, **c** The white arrowheads indicate type Ia endoleak. **d** The maximum diameter of the distal aortic arch is 57 mm. **e** The true lumen compression is improved by thoracic endovascular aortic repair, but the stent graft remains slightly compressed. **f** The white arrow indicates a re-entry located above the celiac artery

A 69-year-old man who presented with acute back pain was referred to our hospital. He had a history of severe chronic obstructive pulmonary disease (COPD) and an abdominal aortic aneurysm treated with EVAR. The patient was diagnosed with acute TBAD with the primary entry located at the distal aortic arch by enhanced computed tomography (CT) (Fig. 1a–c). The patient was admitted and received medical treatment. Forty-eight hours after the onset, follow-up enhanced CT revealed that the true lumen of the descending aorta was severely compressed by the false lumen (Fig. 1d). Four days after the onset, the patient underwent TEVAR using a Zenith Alpha Thoracic System (Cook Medical, Bloomington, IN, USA). First, a distal extension (ZTA-DE-30–108-W1) was deployed in the descending aorta. Second, a proximal device (ZTA-PT-40–36-167-W1) was deployed at the distal aortic arch distal to the left subclavian artery. Although type Ia endoleak was suspected, TEVAR was terminated because the compressed true lumen improved (Fig. 2a). The clinical course of the acute phase after TEVAR was uneventful, and the patient was discharged 10 days after the TEVAR. However, the follow-up CT indicated a gradual increase in the diameter of the distal aortic arch and the descending aorta. Enhanced CT at 10 months after the TEVAR showed that the false lumen remained open with type Ia endoleak and re-entry above the celiac artery, and the maximum diameter of the distal aortic arch enlarged to 57 mm (Fig. 2b–f). Furthermore, marked bleeding tendencies, including several large ecchymoses, and abnormal coagulation were observed. His laboratory data were as follows: hemoglobin, 8.7 g/dL; platelet count, 66,000/μL; fibrinogen degradation product (FDP), 229.0 μg/mL; d-dimer, 164.4 μg/mL; fibrinogen, 80.7 mg/dL; thrombin antithrombin complex (TAT), 86.3 ng/mL; and plasmin-α2 plasmin inhibitor complex (PIC), 9.8 μg/mL. His coagulation time was slightly prolonged; the international normalized ratio of prothrombin time (PT-INR) and activated partial thromboplastin time (APTT) were 1.19 and 45.0 s, respectively. He was diagnosed with DIC based on the International Society on Thrombosis and Hemostasis (ISTH) criteria and the Japanese Society on Thrombosis and Hemostasis (JSTH) criteria (DIC score: ISTH 5 and JSTH 8). His DIC phenotype was diagnosed as an enhanced fibrinolytic type in accordance with Asakura’s criteria [5].

**Fig. 3:**
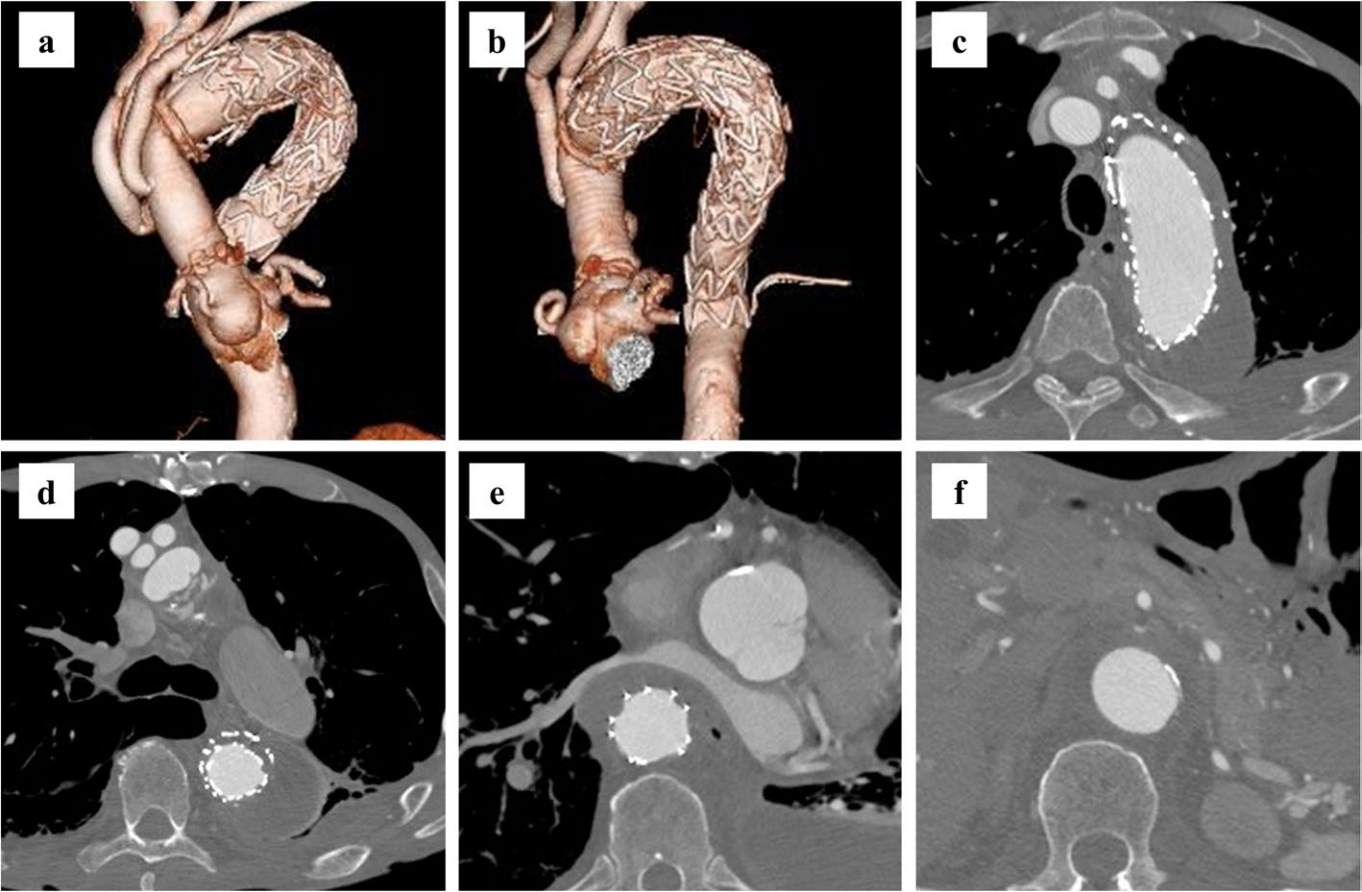
Contrast-enhanced computed tomography at two months after surgery. **a**, **b** Three-dimensional imaging of ascending to descending aorta. **c**, **d** The false lumen is completely thrombosed and shrunk. **e** The compressed stent graft improves. **f** The re-entry above the celiac artery has disappeared, and the false lumen is shrunk

**Fig. 4:**
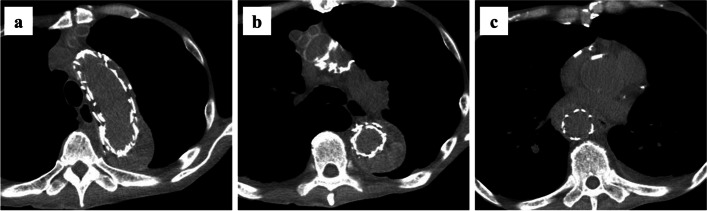
Plain computed tomography at 6 months after the total arch replacement. No obvious change in aortic diameter is observed compared with the previous examination or compression of the stent graft by the false lumen

We considered that additional TEVAR or open conversion was necessary to treat expanded distal aortic arch and endoleak-related enhanced fibrinolytic-type DIC. Zone 2 debranching TEVAR was considered unsuitable because the proximal neck was slightly enlarged. Zone 1 debranching TEVAR, fenestrated TEVAR, or open surgery was discussed. Finally, we considered performing a TAR depending on the patient’s request. Since the bleeding risk of open surgery was considered high, we planned preoperative medical control for DIC. Oral 1500 mg/day tranexamic acid (TXA) and intravenous 12,800 U/day recombinant human soluble thrombomodulin (rTM) were administered. In addition, the patient was transfused with 10 units of platelet concentrates. Immediately before surgery, his plasma FDP, d-dimer, TAT, and PIC concentrations decreased to 14.4 μg/mL, 13.3 μg/mL, 21.5 ng/mL, and 0.9 μg/mL, respectively, whereas his fibrinogen level and platelet count increased to 217.8 mg/dL and 135,000/μL, respectively. Moreover, his coagulation time was normalized as follows: PT-INR, 1.15, and APTT, 36.7 s. TAR was performed 11 months after the TEVAR. Cardiopulmonary bypass was established via venous cannulation of the right atrium and a 9-mm conduit anastomosed to the right axillary and the right femoral arteries. With antegrade cerebral perfusion, the aorta was opened under moderate hypothermic circulatory arrest at a rectal temperature of 25 °C. An open stent graft (OSG; J Graft FROZENIX, FRZX-31150, Japan Lifeline, Tokyo, Japan) was placed in the descending aorta and the Zenith alpha stent graft. A four-branched J graft (Japan Lifeline, Tokyo, Japan) was anastomosed. Postoperatively, he required a massive transfusion during the acute phase. Overall, a total amount of 72 units of red blood cell concentrate, 78 units of fresh frozen plasma, and 100 units of platelet concentrates were transfused during the perioperative period. Oral 1500 mg/day TXA was resumed 10 days after the TAR. On the 14th postoperative day, his platelet levels increased to 159,000/μL, and FDP and d-dimer concentrations decreased to 5.5 μg/mL and 3.2 μg/mL, respectively. The CT 2 months after the TAR revealed that the false lumen at any point in the distal aortic arch to abdominal aorta was completely thrombosed and shrunk (Fig. 3). Despite long-term hospitalization for respiratory complications due to severe COPD and postoperative cholecystitis, he recovered and was transferred for rehabilitation 100 days after the TAR. After he was transferred, oral TXA was continued. The bleeding tendency was not apparent, and his coagulation time remained normal as follows: PT-INR, 1.12, and APTT, 32.3 s. His platelet levels were 153,000/μL, and FDP and d-dimer concentrations were 24.6 μg/mL and 11.7 μg/mL, respectively. After inpatient rehabilitation, he was discharged on postoperative day 156. At 6 months after the TAR, his platelet levels were 101,000/μL, and plain CT revealed no remarkable changes in the aortic diameter or compression of the stent graft by the false lumen (Fig. 4).

## Discussion

Recently, TEVAR for TBAD has become widely accepted, especially in complicated type TBAD. However, TEVAR can cause specific complications, such as endoleak, retrograde type A aortic dissection (RTAD), stent graft migration, and distal stent graft-induced new entry (SINE). A systematic review showed that 15% of patients who received TEVAR for TBAD required reintervention, and 33.2% of the reasons for reintervention were endoleaks [6]. Another study showed that endoleaks were found in 48% of patients who received TEVAR for acute complicated TBAD, of which 17% were type Ia endoleaks [7]. Aortic aneurysms and aortic dissection can cause DIC. Furthermore, DIC due to endoleaks after EVAR has been reported [8, 9].

A possible mechanism of DIC associated with aortic aneurysm is hypothesized that endothelial disruption and turbulent flow in the aortic sac cause exposure of the blood to the denuded endothelium and tissue factors, triggering the coagulation cascade. This leads to the consumption of clotting factors and fibrinolysis of the clots, resulting in DIC. Similarly, in aortic dissection, it is hypothesized that the intima, injured with the formation or expansion of the false lumen, and the turbulent flow in the false lumen expose the denuded endothelium and tissue factors, leading to DIC. In addition, congestion of the blood in the false lumen can form a thrombus, leading to further consumption and liberation of coagulation factors. A possible pathology of endoleak-related DIC could be the injured endothelium and turbulent jet flow induced by the endoleak, similar to aortic aneurysm or dissection [4, 8–11].

DIC can be categorized into three fibrinolytic types: suppressed, enhanced, and balanced. DIC associated with aortic aneurysm and aortic dissection is generally classified as an enhanced fibrinolytic type. This type of DIC is characterized by severe bleeding symptoms; the elevation of TAT, PIC, FDP, and d-dimer (increased FDP/d-dimer ratio); and decreased fibrinogen levels, platelet count, and α2PI activity [3, 5]. Endoleak-related DIC is classified as an enhanced fibrinolytic type because its pathological mechanism is similar to that of aortic aneurysm/dissection. Our patient fulfilled the abovementioned criteria for the diagnosis of enhanced fibrinolytic type DIC.

The cornerstone of DIC treatment is a definitive intervention to address the underlying coagulopathy-causing disorders [1]. Definitive interventions for endoleak-related DIC include the closure of the endoleak. Previous reports have shown that endoleak-related DIC can be treated via the closure of the endoleak with open surgical repair or endovascular procedures [4, 8–11].

In many cases, the specific and radical treatment of the underlying disorder will improve DIC. However, additional supportive treatment, specifically aimed at revising coagulation abnormalities, may be required in some cases. Supportive treatments for DIC include anticoagulant treatments, such as heparin, synthetic protease inhibitors or rTM, blood transfusion, and antifibrinolytic treatments [1]. The treatment with heparin for enhanced-fibrinolytic-type DIC patients is not recommended because of the risk of bleeding [1, 5]. The incidence of bleeding-related adverse events was lower in patients with rTM than in patients with heparin [12]. Another study reported that acute promyelocytic leukemia (APL) patients with enhanced fibrinolytic-type DIC were successfully treated using rTM [13]. Moreover, a retrospective analysis of an open-label, multicenter, post-marketing surveillance study cohort has indicated the safety and efficacy of rTM in patients with APL and DIC [14]. Therefore, we believe that rTM must be effective for DIC caused by aortic aneurysm, aortic dissection, or endoleak. A bleeding complication is a major concern when rTM is used for preoperative treatment, particularly in the cases of open aortic surgery. The bleeding risk due to rTM has been reported to be lower than that of other anticoagulants such as heparin. Previously, it was indicated that preoperative administration of rTM for the case of AAA with DIC improved the DIC score and enabled open surgery without bleeding complications [15].

Antifibrinolytic treatments such as TXA are generally not recommended for patients with DIC. However, patients with enhanced fibrinolytic-type DIC with severe bleeding can be treated with antifibrinolytic agents [1]. Previous reports have suggested that the administration of TXA is useful for bleeding complications of endoleak-related DIC and also contributes to the closure of endoleaks [2, 3]. Previously, we have reported that TXA was effective for DIC associated with aortic dissection or aortic aneurysm[16]. We believe that endoleak-related DIC should be treated by the closure of the endoleak with open surgery or endovascular treatment, if possible. However, not all patients with endoleak-related DIC can be treated endovascularly, and surgical treatment, especially open aortic repair with cardiopulmonary bypass, is accompanied by the risk of massive bleeding for DIC patients. Medications, such as the administration of rTM, TXA, and blood transfusion, may be effective in reducing the risk of perioperative bleeding.

Persistent endoleaks after TEVAR could cause enhanced fibrinolytic-type DIC.

We experienced a case of endoleak-related DIC after TEVAR for TBAD who was successfully treated with TAR and several perioperative medications.

## Data Availability

The datasets used and/or analyzed during the current study are available from the corresponding author upon reasonable request.
